# Effectiveness of life review on depression among elderly: a systematic review and meta-analysis

**DOI:** 10.11604/pamj.2021.40.168.30040

**Published:** 2021-11-18

**Authors:** Bushra Rashid Al-Ghafri, Abdulaziz Al-Mahrezi, Moon Fai Chan

**Affiliations:** 1 Department of Family Medicine and Public Health, College of Medicine and Health Sciences, Sultan Qaboos University, Muscat, Oman

**Keywords:** Life review, elderly, depression, meta-analysis, systematic review

## Abstract

**Introduction:**

depression is considered one of the most common obstacles to daily life activities and quality of life in the elderly. Evidence is accumulating regarding the effectiveness of reminiscence and life review interventions in reducing depression and raising well-being in the elderly. The aim of this review was to determine the effects of life review interventions on depression outcomes among the elderly.

**Methods:**

a search of the literature was performed through 11 electronic databases to identify all randomized controlled trials studies that examine life review effects on depression among the elderly. For each study, the effect size (Cohen's d) between groups (life review vs. control) differences in depression scores for post-intervention and follow-up intervention were computed.

**Results:**

in total, 15 studies were met the inclusion criteria and was evaluated by meta-analysis. Results showed that the life review group has a large effect on reducing depression level than the control group on post-intervention and follow-up. After conducted sensitivity analysis, a moderate effect (effect size=-0.54; 95% CI=-0.71 to -0.36; p<0.05) and small effect (effect size=-0.20; 95% CI=-0.41 to -0.01; p<0.05) were found on post-intervention and follow-up, respectively.

**Conclusion:**

through this systematic review and meta-analysis, the overall results showed a moderate effect to reducing depression levels among the elderly in the life review group after carrying out post-intervention measurements, while in the follow-up the effect was small. This review indicates that life review intervention is one of the options likely to be of benefit for elderly in primary care settings, but further research can be focused on intervention and follow-up durations to obtain long-term effects.

## Introduction

The number of older people in every corner of the world keeps rising, creating a challenging issue of health and well-being. This growing number of the elderly has been termed as the silent revolution [1]. As one ages, his or her physical and mental strengths weaken, leading to physical, mental, and social changes that affect the elderly´s quality of life [2]. Depression is considered one of the most common mental disorders hampering daily life activities and reducing quality of life in the elderly [3-7]. Major depression usually affects the age group of 55 years and above, accounting for 1.8% of the general population, while minor depression occurs in 9.8% [8]. Studies on health care costs have demonstrated that older adults with depression have higher health costs than do their non-depressed counterparts, regardless of the presence of other chronic diseases [9]. Depression can accelerate or exacerbate pain, malnutrition, functional disability [10], and lowered immunity [11]. The elderly with depression are three times more likely to have high blood pressure than those of their age who are not depressed, and they are more likely to develop hip fractures, heart attacks, pneumonia, and other infections [11]. There is strong evidence that reminiscence and life review interventions are effective in reducing depression and raising well-being in the elderly [12,13]. Most of the time, the two terms ‘life review´ and ‘reminiscence´ are used interchangeably, but they may not be properly interpreted.

According to Butler, life review is defined as “a naturally occurring, universal mental process characterized by the progressive return to consciousness of past experiences”. It involves recollecting, evaluating and assigning meaning to positive and negative one´s personal memories [14,15]. Reminiscence is a part of life review and a facilitator of it. Haight and colleagues described it as “a rubric with several different functions and represent different reminiscence phenomena” [16,17]. Life review is the most common approach among other treatments for depression [18] in older adults based on evidence, which is specific for the elderly. It was found through meta-analyses that life review shows important and essential effects on depression and other psychological well-being [14,19]. The studies were heterogeneous in terms of the evaluation elements, the number of sessions, the time, and the methods of motivation to review life. Some studies included verbal stimuli alone, whereas some others used pictures, music, and albums [20]. According to recent meta-analysis studies, additional evidence for long-term effects and follow-up periods (of at least one year) is required. Previous studies only evaluated symptoms of depression but not depression itself as an outcome [21]. As a whole, this study aimed to perform a systematic review, focusing on the effects of life review on depression as an outcome in the elderly by considering the number of sessions, the time, the measuring tools of the outcome, the duration, and other related elements.

## Methods

This systematic review was implemented and written using the preferred reporting items for systematic reviews and meta-analyses (PRISMA) guidelines [22].

**Inclusion and exclusion criteria:** the type of studies included in this review was randomized controlled trials (RCT) and the type of intervention was any form of life review compared to no treatment or control. The participants of interest were the elderly at the age of 60 years or older with depression as the outcome. The search limits included full-text articles with available abstracts in human science, general medical, psychiatry, education, counseling, biography and autobiography, social scientists, and psychologists. This review excluded the observational studies and other types of studies (e.g. survey, reviews, and study protocol). We also excluded studies that included participants with Dementia or Alzheimer disease or undergoing other types of intervention (e.g. music therapy, yoga, or cognitive therapy). In addition, articles published from books, e-books, newspapers, and magazines were excluded too.

**Search strategy:** literature study was done by collecting the relevant data from the library of the Sultan Qaboos University. The key words used in the search engine were the terms as “life story”, “storytelling”, “reminiscence”, “reminisce”, or “life review”, and “depression”, “older adults”, ”elderly”, “geriatric”, ”geriatrics”, ”aging”, “senior”, “seniors”, or “older people”. Within the limits of the research were articles published in English and Arabic languages between 1981 and March 2021.

**Data sources:** the articles were collected from the following databases: embase medline, academic search ultimate, CINAHL Plus, CAB Abstract, ERIC, Agricola, Cochrane Library, Green file, Science Direct, Google Scholar, and Springer.

**Data extraction and quality assessment:** data were extracted from articles included in this review with the following information: author´s name, year of publication, country, the total number of samples, the content of intervention and control groups, numbers of females and males, age, sessions period and time (in weeks), dropout, outcome measuring tool, form of delivery (individual/group) and results (during post-intervention and follow-up periods). Post-intervention is the period of taking outcomes after applying intervention immediately, while in the follow-up the outcomes were measured after a period of time spanning several weeks after finishing the intervention. According to the Joanna Briggs Institute (JBI) critical appraisal checklist for systematic reviews [23], the obtained full-text articles were evaluated to estimate the quality of each study. The initial search and screening the title and abstract was conducted by one researcher (BRG). A full-text was conducted by two researchers (BRG and MFC) to assess the eligibility. One researcher (AM) was consulted whenever a disagreement arose between other two researchers (BRG and MFC) but there were no discrepancies. At the end, a total of 15 studies were included in the final analysis.

**Statistical analysis:** descriptive statistics (e.g. mean, standard deviation, frequency) were used to describe the characteristic of each reviewed study. Meta-analysis was used to integrate all reviewed studies and estimate the overall effect sizes of the life review against control [24] on depression level. Depression score is the outcome measure of each reviewed study. All reviewed studies, including the number of samples, mean, standard deviation (SD) on depression of the life review and control groups were extracted. For each study, the effect size (Cohen´s d) between groups (life review vs. control) differences in depression scores for post intervention and follow-up intervention were computed. An effect size interpreted as small (0 to 0.32), moderate (0.33 to 0.55), or large (0.56 or higher) according to previous studies [25,26]. I^2^ and Cohran´s Q statistics were used to assess homogeneity across studies [27] for post-intervention and follow-up intervention. Random-effects model was used to calculated the pooled effect size for after intervention and follow-up because the I^2^ 50% and the p-value on Q statistic < 10% were shown as heterogeneity of the reviewed articles. Since, there was a significant heterogeneity across studies, sensitivity analysis was conducted to identify the presence of publication bias by using funnel plot and Egger's test. The acquired data were analyzed using the MedCalc 12 statistical software.

## Results

**Figure 1:**
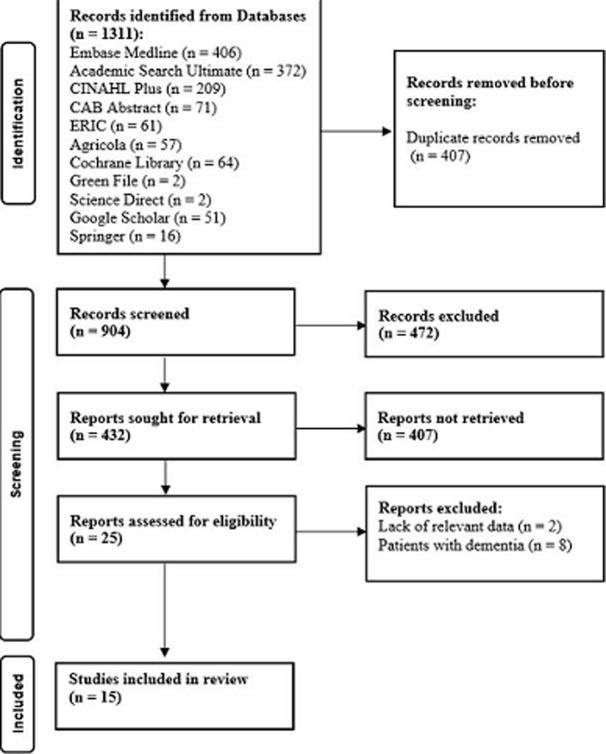
publication selection flow chart

**Study selection:** one thousand three hundred and eleven abstracts were obtained in the first search, out of which 407 duplicates were removed, leaving 904 results. Furthermore 472 irrelevant publications were excluded, therefore there were only 432 sought for retrieval, while 25 remained for screening for the title and abstract by looking at the full-text articles for eligibility assessment; 8 studies were excluded because they failed to meet the inclusion criteria, and another 2 were excluded due to lack of relevant data. Thus, 15 studies were included in this review and analyzed according to the Intensive Behavioural Intervention (IBI) critical appraisal checklist for systematic reviews. The publication selection flow chart showed the specific outcomes and causes (Figure 1).

**Studies quality:** in all studies, participants were randomly assigned, and allocation to groups was concealed. Regarding the similarity of the treatment groups at baseline, the difference was only found in two studies. One of which [28] showed no similarity, while the other [29] remained unclear. In addition, only one study [29] showed that the participants were blind to treatment assignment. Besides this, the same study and two other studies [30,31] showed that the treatment assignment was hidden from the treatment providers. Regarding follow-up completion, two studies [28,29] were unclear on this aspect. One study [30] also remained unclear for the relevance of trial design to the topic. The quality assessment results of the included studies are shown in Table 1.

**Table 1:** quality of included randomized controlled trial (RCT) studies in the systematic review using JBI RCT checklist

Article	Q1	Q2	Q3	Q4	Q5	Q6	Q7	Q8	Q9	Q10	Q11	Q12	Q13
Chan, 2013	Y	Y	Y	N	N	N	Y	Y	Y	Y	Y	Y	Y
Chan, 2014	Y	Y	Y	N	N	N	Y	Y	Y	Y	Y	Y	Y
Haight, 1992	Y	Y	N	Y	Y	N	Y	U	Y	Y	Y	Y	Y
Hanaoka, 2004	Y	Y	Y	N	N	N	Y	Y	Y	Y	Y	Y	Y
IIali, 2019	Y	Y	Y	N	N	N	Y	Y	Y	Y	Y	Y	Y
Korte, 2011	Y	Y	Y	N	N	N	Y	Y	Y	Y	Y	Y	Y
Latorre, 2014	Y	Y	Y	N	N	N	Y	Y	Y	Y	Y	Y	Y
Mastel-Smith, 2007	Y	Y	Y	N	N	N	Y	Y	Y	Y	Y	Y	Y
Pot, 2010	Y	Y	Y	N	N	N	Y	Y	Y	Y	Y	Y	Y
Preschl, 2012	Y	Y	Y	N	N	N	Y	Y	Y	Y	Y	Y	Y
Sabir, 2015	Y	Y	Y	N	N	N	Y	Y	Y	Y	Y	Y	Y
Serrano, 2004	Y	Y	Y	N	Y	N	Y	Y	Y	Y	Y	Y	Y
Serrano, 2012	Y	Y	Y	N	Y	N	Y	Y	Y	Y	Y	Y	Y
Shellman, 2009	Y	Y	Y	N	N	N	Y	Y	Y	Y	Y	Y	Y
Stevens-Ratchford, 1993	Y	Y	Y	N	N	N	Y	Y	Y	Y	Y	Y	Y

Y:yes; N:no; U: unclear; NA: not available; JBI: Joanna Briggs Institute; Q1: was true randomization used for the assignment of participants to treatment groups?; Q2: was allocation to treatment groups concealed?; Q3: were treatment groups similar at the baseline?; Q4: were participants blind to treatment assignment?; Q5: were those delivering treatment blind to treatment assignment?; Q6: were outcomes assessors blind to treatment assignment?; Q7: were treatment groups treated identically other than the intervention of interest?; Q8: were follow-up complete and if not, were differences between groups in terms of their follow-up adequately described and analyzed?; Q9: were participants analyzed in the groups to which they were randomized?; Q10: were outcomes measured in the same way for treatment groups?; Q11: were outcomes measured reliably?; Q12: was appropriate statistical analysis used?; Q13: was the trial design appropriate, and any deviations from the standard RCT design (individual randomization, parallel groups) accounted for in the conduct and analysis of the trial?

**Studies characteristics:** the number of studies included in this review was 15 with a total of 963 participants, among which there were five studies conducted in the USA, three in Spain, two in Singapore as well as in the Netherlands, and for the rest, only one study was conducted in each of the following countries: Japan, German and Iran. The studies varied in the number of sessions they included, two studies [31,32] contained five sessions, two studies [29,30] contained four sessions, five studies [28,33-36] included six sessions, and four studies [20,37-40] included eight sessions. The other two studies contained 10 sessions [41] and 12 sessions [42]. The contents of stimulating intervention to review life differed, including life review story, writing, art-based life review, remembering specific positive events, computer supplements, and autobiographical retrieval practice. All of the interventions took place individually except in two studies [35,38], which were in a group form. Depression levels were measured using different screening instruments such as a 20 item self-report scale (CES-D; 6 studies), geriatric depression scale-15 (GDS-15; 3 studies) or scale-30 (GDS-30; 2 studies), beck depression inventory-II (BDI-II; 2 studies), a screening instrument consists of three subscales: depression, somatization and anxiety (BSI-18; 1 study) and Zung´s depression scale (SDS; 1 study). Further details on the characteristics of the studies are shown in Table 2.

**Table 2:** main characteristics of the randomized controlled studies included in the systematic review

Author, Year	Country	N	Content I / C	F/M	Age M (SD)	Sessions (period) / Wk	DO	Tool	Form	Results wk / n / M (SD) / p-value [PI/FU]
Chan, 2013	Singapore	26	I: LSR+DSB/C: NI	21/5	69.7(6.8)	5 (30-45 m ) / 8	0	GDS-15	Ind	w0: I: n=14 / 7.9 (3) vs. C: n=12 / 6.3 (2.5) / p=0.157 w4: I: n=14 / 4.6 (1.9) vs. C: n=12 / 5.4 (2.5)/ p=0.058 [PI] w8: I: n=14 / 2.5 (1.7) vs. C: n=12 / 5.3 (2.1) / p <.0.001 [FU]
Chan, 2014	Singapore	29	I: LSR / C: NI	23/6	68.97(6.46)	5 (30-45 m) / 8	0	GDS-15	Ind	w0: I: n=15 / 5.9 (2.3) vs. C: n=14 / 5.0 (1.3)ï¿½ / p=0.210 w4: I: n=15 / 2.5 (2.2) vs. C: n=14 / 2.6 (1.4) / p<0.001 [PI] w8: I: n=15 / 1.9 (1.6) vs. C: n=14 / 3.5 (1.5) / p =.0.001 [FU]
Haight, 1992	USA	51	I: LR/C: NI/V: NT*	40/11	76(NA)	6 (1 hr) / 52	16	SDS	Ind	w0: I: n=10 / 25 (8.43) vs. C: n=12 / 27.3(13) / p=0.636 w8: I: n=10 / 17.3 (7.8) vs. C: n=12 / 24.2(9.3) / p=0.077 [PI] w52: I: n=10 / 17.2 (8.4) vs. C: n=12 / 16.6(8.64) / p=0.871 [FU]
Hanaoka, 2004	Japan	80	I: LRA / C: NI	69/11	I:81.62; C:81.97	8 (1 hr) / 20	9	GDS-30	Ind	w0: I: n=42 / 13.56 (5.94) vs. C: n=38 / 13.57 (6.57) / p= 0.730 w8: I: n=40 / 13.67 (3.04) vs. C: n=36 / 11.83 (4.23) / p=0.390 [PI] w20: I: n=36 / 12.73 (4.74) vs. C: n=35 / 13.34 (3.57) / p=0.040 [FU]
IIali, 2019	Iran	58	I: ABLR / C: NI	32/22	70(NA)	6 (1 hr) / 6	4	GDS-15	Ind	w0: I: n=27 / 4.333 (2.401) vs. C: n=27 / 4.703 (2.825) / p=0.606 w2: I: n=27 / 2.185 (2.076) vs. C: n=27 / 4.703 (2.958) / p=0.0007 [PI] w6: I: n=27 / 1.444 (1.671) vs. C: n=27 / 5.407 (2.692) / p=0.001 [FU]
Korte, 2012	Netherland	202	I: LRT/ C: NI	155/47	63.3(6.5)	8 (2 hr) / 24	0	CES-D	Grp	w0: I: n=100 / 20.5 (1.1) vs. C: n=102 / 20.6 (0.74) / p=0.449 w12: I: n=100 / 15.8 (1.2) vs. C: n=102 / 21.2 (0.90) / p <.0.001 [PI] w24: I: n=100 / 15.3 (1.1) vs. C: n=102 / 20.4 (1.0) / p <.0.001 [FU]
Latorre, 2014	Spain	55	I: LR+RSPE/C: MW	18/37	65.35(8.45)	6 (NA) / 8	0	CES-D	Grp	w0: I: n=29 / 12.66 (9.37) vs. C: n=26 / 10 (8.14) / p=0.270 w8: I: n=29 / 8.14 (5.58) vs. C: n=26 / 12.12 (9.75) / p=0.005 [PI]
Mastel-Smith, 2007	USA	33	I: LR+W / C: NI	27/6	70.12(6.83)	10 (2 hr) / 11	2	BSI-18	Ind	w0: I: n=16 / 44.47 (5.59) vs. C: n=17 / 44.94 (6.31) / p=0.830 w11: I: n=15 / 42.60 (3.07) vs. C: n=16 / 47.81 (8.29) / P = 0.036 [PI]
Pot, 2009	Netherland	171	I: LR / C: VW	124/47	64.3(7.4)	12 (2 hr) / 36	25	CES-D	Ind	w0: I: n=83 / 21.31 (7.68) vs. C: n=88 / 20.07 (7.59) / p =0.290 w12: I: n=79 / 14.97 (7.40) vs. C: n=74 / 18.17 (8.95) / p =0.01 [PI] w36: I: n=78 / 15.12 (8.34) vs. C: n=68 / 17.03 (8.71) / p= 0.15 [FU]
Presch, 2012	German	36	I: LRT+CS/C: NI	24/12	70.0 (4.4)	6 (11.5 hr) / 8	0	BDI-II	Ind	w0: I: n=20 / 19 (6.6) vs. C: n=16 / 16.5 (5.6) / p=0.236 w8: I: n=20 / 10 (6.3) vs. C: n=16 / 15.1 (7.8) / p <.0.01 [PI] w20: I: n=14 / 8.7 (4.8) vs. C: NA [FU]
Sabir, 2015	USA	62	I: IR / C: NI	56/6	72(8)	8 (2 hr) / 32	1	CES-D	Ind	w0: I: n= 32 / 19.79( 14.08) vs. C: n= 29 / 14.67 (13.16) / p=0.01 w8: I: n= 32 / 16.19 (14.76) vs. C: n=29 / 16.50 (13.53) / p=0.05 [PI] w32: I: n= 32 / 14.93 (14.80) vs. C: n=29 / 14.10 (13.96) / p=0.05 [FU]
Serrano, 2004	Spain	43	I: LRT+ARP/C: NI	33/10	77.19 (7.68)	4 (NA) / 8	0	CES-D	Ind	w0: I: n=20 / 30.70 (6.76) vs. C: n=23 / 27.61 (6.29) / p=0.128 w8: I: n=20 / 20.45 (7.25) vs. C: n=23 / 27.61 (7.48) / p <0.001 [PI]
Serrano, 2012	Spain	37	I: LRT+ARP/C: NI	31/6	73.9(NA)	4 (1 hr) / 28	20	GDS-30	Ind	w0: I: n=18 / 17.3 (5.2) vs. C: n=19 / 22.5 (3.2) / p<0.05 w4: I: n=13 / 14.1 (9.8) vs. C: n=13 / 18.5 (7.4) / p=0.027 [PI] w10: I: n=12 / 13.1 (8.8) vs. C: n=14 / 14.8 (6.0) / p=0.566 [FU]
Shellman, 2009	USA	56	I: IR/AC: HC**/C: NI	43/13	72.6(8.6)	8 (45 m) / 8	0	CES-D	Ind	w0: I: n=19 / 9.9 (5.3) vs. C: n=18 / 11.3(12.5) / p=0.657 w8: I: n=19 / 6.8 (4.7) vs. C: n=18 / 14.6(10.8) / p= 0.001 [PI]
Stevens-Ratchford,1993	USA	24	I: LRRA / C: NI	16/8	79.75(NA)	6 (2 hr) / 4	0	BDI	Ind	w0: I: n=12 / 26.58 (4.7) vs. C: n=12 / 30.17 (7.2) / p=0.162 w4: I: n=12 / 25.45 (4.5) vs. C: n=12 / 28.83 (6.5) / p = .695 [PI]

N: total samples; LR: life review; LRT: life review therapy; LRA: life review activities; LSR: life story review; ARP: autobiographical retrieval practice; IR: integrative reminiscence; F/M: female/ male; Grp: Group; wk: week; LRRA: life review reminiscence activities; ACTP-LR: a culturally tailored peer-led reminiscence; NI: no intervention; DSB: develop story book; W: writing; I: intervention group; C: control group; Ind: individual; VW: video watch; AC: attention control; PI: post-intervention measure; FU: follow-up measure; M (SD): mean (Standard deviation); V: visit; NA: not Available; hr: hour; m: minutes; SDS: Zungs depression Scale; MC: mean change; CES-D: center for epidemiologic studies depression scale; BDI-II, beck depression inventory-II; GDS-15/-30, geriatric depression Scale-15/-30;*: visit group was not shown; ABLR: art-based life review; NA: Not available; **: attention control group was not shown; MW: media workshop; RSPE: remembering specific positive events; CS: computer supplements; HE: health education; Form: format

**Figure 2:**
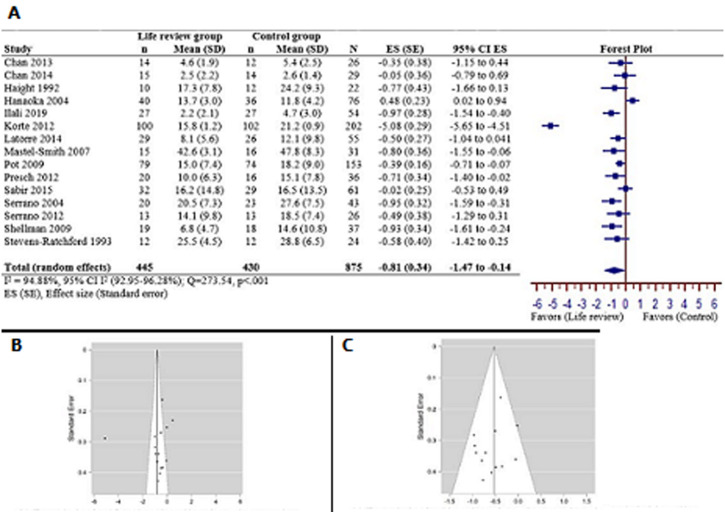
A) forest plot of the meta-analysis for studies of life review group versus the control group of post-intervention on depression rating scores; B) funnel plot for 15 reviewed studies of post-intervention; C) plot for 13 studies of post-intervention after excluding 3 studies (Hanaoka 2004, Korte 2012)

**Post-intervention effects:** the meta-analysis in Figure 2A displays the post-intervention effects on 15 studies [21,29,30-42] of the life review compared to a control condition. The pooled data included 875 samples (life review=445 vs. control=430), and its heterogeneity was high (I^2^=94.88%) and significant (Q=273.54; p<0.001). Therefore, the random effects model was used, and it showed that the life review groups had a larger effect on reducing depressive symptoms (pooled effect size=-0.8; 95% CI=-1.47 to -0.14; p<0.05) than the control group. Publication bias was noted in a few studies from the funnel plot (Figure 2B). Sensitivity analysis was performed and suggested that 2 studies [21,38] should be excluded (Egger´s test: Z=1.109, p=0.268) and its heterogeneity was changed to low (I^2^=10.978%) and not significant (Q=13.480, p=0.335) (Figure 2C). Therefore, a fixed effects model was used for these 13 articles [29,30-37,39-42] and a moderate effect size was found (pooled effect size=-0.54, 95% CI -0.71 to -0.36).

**Figure 3:**
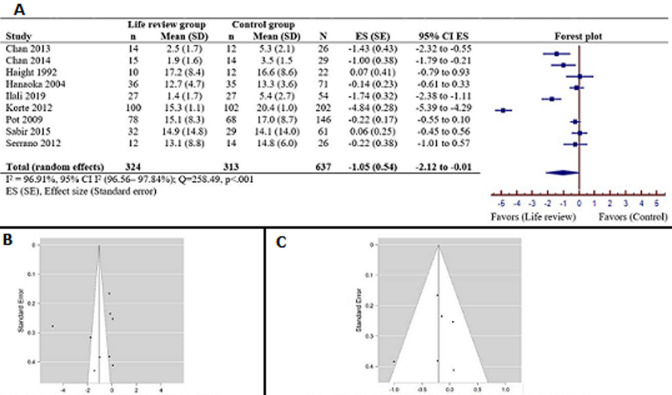
A) forest plot of the meta-analysis for studies of life review group versus control group of follow-up on depression rating scores; B) funnel plot for 9 reviewed studies of follow-up; C) funnel plot for 6 studies of follow-up after excluding 3 studies (Chan 2013, Ilali 2019, Korte 2012)

**Follow-up effects:** the meta-analysis in Figure 3A displays the follow-up effects in 9 studies [21,29,31-34,38,39,42] of the life review compared to a control condition. The pooled data included 637 samples (life review=324 vs. control=313) and its heterogeneity was high (I^2^=96.91%) and significant (Q=258.49; p<0.001). Therefore, the random effects model was used, and it showed that the follow-up effects on life review groups had a larger effect on reducing depressive symptoms (pooled effect size=-1.05; 95% CI=-2.12 to -0.01; p<0.05) than the control group. Publication bias was noted in a few studies from the funnel plot (Figure 3B). Sensitivity analysis was performed and suggested that 3 studies [32,34,38] should be excluded (Egger´s test: Z=0.526, p=0.599) and its heterogeneity was changed to low (I^2^=15.196%) and not significant (Q=5.896, p=0.316) (Figure 3C). Therefore, a fixed effects model was used for these 6 articles [21,29,31,33,39,42] and a small effect size was found (pooled effect size=-0.20, 95% CI -0.41 to -0.01).

## Discussion

The objective of this review was to determine the effectiveness of life review on depression among elderly. The results of the included studies reported a significant reduction of depression scores in the intervention group over time. Meta-analysis results showed that the life review groups had a large effect on reducing depressive symptoms than the control groups in both post-intervention and follow-up periods when the random effects model was used. Whereas, the sensitivity analysis showed that life review had a moderate effect on reducing depressive symptoms in post-intervention and a small effect in the follow-up. With regard to the duration of data collection, we found that it ranged between 2 and 12 weeks, in which 8 weeks was the most common. All studies began to record the initial measurements at baseline, and the measurement periods varied afterward, as some studies were limited to the post-intervention period and some included follow-up after one, three, six, nine months, or one year. The results also showed that the dropout rate was greater in studies that included follow-up periods of longer duration.

By comparing studies related to the title of this review, our findings in terms of a positive effect of life review in reducing depression levels are consistent with the results found in 2003 by Hseih and Wang which stated that reminiscence therapy resulted in statistical significantly decrease in depression in elderly [42], and the explanation was given by Cappeliez *et al*. (2006) when they found that supporting the elderly by listening to their experiences and confessions and confirming their accomplishments helps them reformulate thoughts and reduce feelings of regret and frustration, and every negative thing associated with depression [43]. In addition, regarding duration of data collection on follow-up which lasted in 8 weeks for most of the studies, this reinforces the conclusion of Haight and Haight in 2007 [44] that a minimum duration of 6-8 weeks of data collection was better for older adults to gain the benefits from life review. This has been explained by Lan *et al*. (2017) that the reason may be that the reviewer and the listener need enough time to gain trust and build a relationship that allows for discussing the details of life [45]. In case of long duration in follow-up periods, the causes of high dropout rate varied between fatigue, the burden of symptoms, or death. This might explain the lack of studies with long-term effects and follow-up periods in this area.

The meta-analysis focused on how effective the life review on depressive symptoms to the elderly on post-intervention and follow up compared with the control group. Initially, 15 and 9 studies were used to identify the pooled effects of life review on depression level for elderly at post-intervention and follow-up, respectively. Significant heterogeneity results were found in both periods, so sensitivity analysis was performed to identify studies with high publication bias that induces such heterogeneity. After removing those highly heterogenous studies, 13 and 6 studies showed homogeneity on post-intervention and follow-up, respectively. A moderate effect on reducing depression level was found on post-intervention which are similar to Lan *et al*. (2017) [45] but a large effect was found from Westerhof and Slatman (2019) [22]. In addition, Westerhof and Slatman (2019) reported a moderate effect size on the follow-up, but a small effect size was found in our study. One of the reasons may be due to the number of weeks; while Westerhof and Slatman (2019) focused on studies of 12 weeks duration only, the duration of the studies included in this review ranged from 6 weeks to 52 weeks. This finding might indicate that follow-up time is a moderating factor that affects the depressive symptoms in the elderly. Another reason for the loss of effects at follow-up may be due to the smaller number of available studies that provided follow-up data.

The strength of this review is that it reinforces previous research confirming the effectiveness of life review in improving depression levels in elderly. It also gathers studies varied in locations as they were conducted in different countries, leading to diversity in environments and cultures, which means that the elderly who suffer from depression may have different beliefs which highlights a new point to focus on for future search and makes these findings stronger. The review also had some limitations: the search for studies was limited to English and Arabic only. There may be other studies conducted in different languages which were not included. The variation in the content of the intervention methods (e.g. life story review, reminiscence, life review therapy) and tools of outcome measurement among studies leads to determining which methods or measures were the best to enhance the review of life or make it more effective. It may be better for future research to focus on one type of intervention and on a specific scale to determine the most effective. This study focuses on only depression as an outcome could be considered a limitation, as life review could impact on other psychotically aspects (e.g. meaning in life) early on that affect their quality of life. Besides, the findings cannot be generalized to other countries than those in which the included studies were conducted, particularly for the Arab countries where the culture and the health care systems are different from the Western or Asian countries. This provides another area for future research by focusing this type of research on Arab countries.

## Conclusion

Through this systematic review and meta-analysis, we aimed to provide healthcare professionals with a comprehensive summary of the available evidence on the effects of life review interventions on depression levels among the elderly. Despite a large variation in the sample size, contents and duration of the intervention, measurement tools, and publication bias, the overall results showed a moderate effect to reducing depression levels among the elderly in the life review group after carrying out post-intervention measurements, while in the follow-up the effect was small. This review indicates that life review intervention is one of the options likely to be of benefit for elderly in primary care settings, but further research can focus on intervention and follow-up durations to obtain long-term effects.

### What is known about this topic

- Depression is considered as one of the most common obstacles to daily life activities and having a good quality of life in the elderly;
- Life review interventions are effective in reducing depression in elderly on post-intervention but the effect is unclear on the follow-up.

### What this study adds

- Life review intervention has a moderate effect to reducing depression levels among the elderly on post-intervention, while in the follow-up the effect was small;
- The reviewed studies varied in specific protocols used and the delivery format but all of them included life review as an intervention;
- Duration of data collection during follow-up period can affect the depressive symptoms in the elderly.

## References

[1] Hesamzadeh A, Maddah SB, Mohammadi F, Fallahi KM, Rahgozar M (2010). Comparison of elderlys” quality of life” living at homes and in private or public nursing homes. Iranian Journal of Ageing.

[2] Senol V, Unalan D, Soyuer F, Argun M (2014). The relationship between health promoting behaviors and quality of life in nursing home residents in Kayseri. Journal of Geriatrics.

[3] Blazer DG (2003). Depression in late life: review and commentary. J Gerontol A Biol Sci Med Sci.

[4] Jang Y, Bergman E, Schonfeld L, Molinari V (2006). Depressive symptoms among older residents in assisted living facilities. Int J Aging Hum Dev.

[5] Li LW, Conwell Y (2009). Effects of changes in depressive symptoms and cognitive functioning on physical disability in home care elders. J Gerontol A Biol Sci Med Sci.

[6] Penninx BW, Leveille S, Ferrucci L, van Eijk JT, Guralnik JM (1999). Exploring the effect of depression on physical disability: Longitudinal evidence from the established populations for epidemiologic studies of the elderly. Am J Public Health.

[7] Watson LC, Lehmann S, Mayer L, Samus Q, Baker A, Brandt J (2006). Depression in assisted living is common and related to physical burden. Am J Geriatr Psychiatry.

[8] Beekman ATF, Copeland JR, Prince MJ (1999). Review of community prevalence of depression in later life. Br J Psychiatry.

[9] Katon W, Lin E, Russo J, Unutzer J (2003). Increased medical costs of a population-based sample of depressed elderly patients. Arch Gen Psychiatry.

[10] Katon W (1996). The impact of major depression on chronic medical illness. Gen Hosp Psychiatry.

[11] McGuire L, Kiecolt-Glaser JK, Glaser R (2002). Depressive symptoms and lymphocyte proliferation in older adults. J Abnorm Psychol.

[12] Bohlmeijer E, Kramer J, Smit F, Onrust S, van Marwijk H (2009). The effects of integrative reminiscence on depressive symptomatology and mastery of older adults. Community Ment Health J.

[13] Bohlmeijer E, Roemer M, Cuipers P, Smit F (2007). The effects of reminiscence on psychological well-being in older adults: a meta analysis. Aging Ment Health.

[14] Butler RN (1963). The life review: an interpretation of reminiscence in the aged. Psychiatry.

[15] Butler RN (1974). Successful aging and the role of life review. J Am Geriatr Soc.

[16] Haight B, Webster J (1995). The art and science of reminiscing: theory, research, and applications. Taylor & Francis.

[17] Webster J, Haight B (2002). Critical advances in reminiscence work from theory to application. Springer Publishing Company.

[18] Scogin F, Welsh D, Hanson A, Stump J, Coates A (2005). Evidence-based psychotherapies for depression in older adults. Clinical Psychology Science Practice.

[19] Bohlmeijer E, Smith F, Cuipers P (2003). Effects of reminiscence and life review on later-life depression: a meta-analysis. Int J Geriatr Psychiatry.

[20] Hanaoka H, Okamura H (2004). Study on effects of life review activities on the quality of life of the elderly: a randomized controlled trial. Psychother Psychosom.

[21] Westerhof GJ, Slatman S (2019). In search of the best evidence for life review therapy to reduce depressive symptoms in older adults: a meta-analysis of randomized controlled trials. Clinical Psychology: Science and Practice.

[22] Moher D, Libereti A, Tetzlaff J, Altman DG, Prisma Group (2009). Preferred reporting items for systematic reviews and meta-analyses: the PRISMA statement. PLoS medicine.

[23] Aromataris E, Munn Z (2020). Jonna briggs institute manual for evidence synthesis. JBI.

[24] Petrie A, Bulman JS, Osborn JF (2003). Further statistics in dentistry Part 8: systematic reviews and meta-analyses. Br Dent J.

[25] Lipsey MW, Wilson DB (1993). The efficacy of psychological, educational, and behavioral treatment: confirmation from meta-analysis. Am Psychol.

[26] Higgins JP, Thompson SG, Deeks JJ, Altman DG (2003). Measuring inconsistency in meta-analyses. BMJ.

[27] DerSimonian R, Laird N (1986). Meta-analysis in clinical trials. Control Clin Trials.

[28] Haight BK (1992). Long-term effects of a structured life review process. J Gerontol.

[29] Serrano JP, Latorre JM, Gatz M, Montanes J (2004). Life review therapy using autobiographical retrieval practice for older adults with depressive symptomatology. Psychol Aging.

[30] Serrano JP, Latorre JM, Ros L, Navarro B, Aguilar MJ, Nieto M, Gatz (2012). Life review therapy using autobiographical retrieval practice for older adults with clinical depression. Psicothema.

[31] Chan MF, Ng SE, Tien A, Man Ho RC, Thayala J (2013). A randomised controlled study to explore the effect of life story review on depression in older Chinese in Singapore. Health Soc Care Community.

[32] Chan MF, Leong K SP, Heng BL, Mathew BK, Khan SB AL, Loudusamy SS (2014). Reducing depression among ommunity-dwelling older adults using life-story review: a pilot study. Geriatr Nurs.

[33] IIali ES, Mokhtary F, Mousavinasab N, Tirgari (2018). Impact of art-based life review on depression symptoms among older adults. Art Therapy.

[34] Latorre JM, Serrano JP, Ricarte J, Bonete B, Ros L, Sitges E (2015). Life-review based on remembering specific positive events in active aging. J Aging Health.

[35] Preschl B, Maercker A, Wagner B, Forstmeier S, Baños RM, Alcañiz M al (2012). Life-review therapy with computer supplements for depression in the elderly: A randomized controlled trial. Aging Ment Health.

[36] Stevens-Ratchford RG (1993). The effect of life review reminiscence activities on depression and self-esteem in older adults. Am J Occup Ther.

[37] Korte J, Bohlmeijer ET, Cappeliez P, Smit F, Westerhof GJ (2012). Life review therapy for older adults with moderate depressive symptomatology: a pragmatic randomized controlled trial. Psychol Med.

[38] Sabir M, Henderson CR, Kang SY, Pillemer K (2016). Attachment-focused integrative reminiscence with older African Americans: a randomized controlled intervention study. Aging Ment Health.

[39] Shellman J, Mokel M, Hewitt N (2009). The effects of integrative reminiscence on depressive symptoms in older African Americans. West J Nurs Res.

[40] Mastel-Smith BA, McFarlane J, Sierpina M, Malecha A, Haile B (2007). Improving depressive symptoms in community-dwelling older adults: a psychosocial intervention using life review and writing. J Gerontol Nurs.

[41] Pot AM, Bohlmeijer ET, Onrust S, Melenhorst AS, Veerbeek M, De Vries W (2010). The impact of life review on depression in older adults: a randomized controlled trial. Int Psychogeriatr.

[42] Hseih H, Wang J (2003). Effect of reminiscence on depression in older adults: a systematic review. Int J Nurs Stud.

[43] Cappeliez P, O´Rourke N (2006). Empirical validation of a model of reminiscence and health and later life. J Gerontol B Psychol Sci Soc Sci.

[44] Haight BK, Haight BS (2007). The handbook of structured Life review. Health Professions Press.

[45] Lan X, Xiao H, Chen Y (2017). Effects of life review intervention on psychosocial outcomes among older adults: a systematic review and meta-analysis. Geriatr Gerontol Int.

